# Secondary prevention of coronary heart disease in Aboriginal and Torres Strait Islander people in primary care

**DOI:** 10.1111/imj.70025

**Published:** 2025-03-24

**Authors:** Edwina Wing‐Lun, Simone Marschner, Desi Quintans, Sean Taylor, Jakelin Troy, Clara Chow, Sarah Zaman

**Affiliations:** ^1^ Westmead Applied Research Centre, Faculty of Medicine and Health University of Sydney Sydney New South Wales Australia; ^2^ Menzies School of Health Research Charles Darwin University Darwin NT Australia; ^3^ Department of Cardiology Royal Darwin Hospital Darwin Australia; ^4^ Onemda: Aboriginal and Torres Strait Islander Health and WellBeing The University of Melbourne Melbourne Victoria Australia; ^5^ Faculty of Arts and Social Sciences University of Sydney Sydney New South Wales Australia; ^6^ Department of Cardiology Westmead Hospital Sydney New South Wales Australia

**Keywords:** coronary heart disease, Aboriginal and Torres Strait Islander People, primary care, cardiovascular risk factors

## Abstract

**Background:**

Coronary heart disease (CHD) is the primary cause of mortality in Australia and the largest contributor to the ‘gap’ in cardiovascular disease deaths between Aboriginal and Torres Strait Islander (First Nations) people and non‐indigenous Australians.

**Aim:**

To assess secondary prevention of CHD in First Nations people in primary care in Australia.

**Methods:**

Retrospective cohort study of patients with CHD under active primary care management using electronic medical records from 406 general practices across Australia. Ultimately, 50 088 people with CHD were included in the study, and 3.5% of those were First Nations people. After 5.9 years (standard deviation 5.0) in primary care adjusting for gender, age, remoteness, comorbidities, smoking status and continuity of care, First Nations peoples received equal statin (adjusted odds ratio (aOR): 0.9; 95% CI:0.8–1.1, *P* = 0.28), angiotensin‐converting enzyme inhibitors/angiotensin II receptor antagonists (aOR:1.0; 95% CI:0.9–1.2, *P* = 0.85) and beta blockers (aOR:0.9;95% CI:0.8–1.1, *P* = 0.41) prescriptions. First Nations peoples were more likely to achieve BP <1.8 in similar proportions (35.2% vs 36.9%, *P* = 0.16) but less likely to have HDL‐C >1.0 mmol/L (57.5% vs 73.7%, *P* < 0.001), triglycerides<2.0 mmol/L (61.7% vs 76.0%, *P* < 0.001) and HbA1C ≤ 53 mmol/mol (7.0%) (67.7% vs 82.1%, *P* < 0.001). A higher proportion of First Nations people had HbA1c measured (75.7% vs 66.6%, *P* < 0.001).

**Conclusion:**

First Nations peoples with CHD under active primary care management received similar secondary prevention medications and achieved BP and LDL‐C targets as frequently as non‐indigenous Australians. A focus on easier access to facilitate attending primary care is needed to close the gap as well as addressing social determinants of health and structural inequities.

## Introduction

Coronary heart disease (CHD) is the primary cause of mortality in Australia and is the largest contributor to the ‘gap’ in cardiovascular disease deaths between Aboriginal and Torres Strait Islander people and non‐indigenous Australians.^1^ Research shows that Aboriginal and Torres Strait Islander people have a three times higher rate of major coronary events and a 1.4 times higher out‐of‐hospital death rate from CHD compared to non‐indigenous Australians.^1^ This gap is multifactorial and has been variously attributed to differences in risk factors and underlying comorbidities, alongside gaps in care, including primary prevention, in hospital treatment and prescription of secondary preventive medications.^2^

Past research revealed that Aboriginal and Torres Strait Islander people have a more atherogenic lipid profile characterised by low levels of high‐density lipoprotein (HDL)‐cholesterol levels and elevated small low‐density lipoprotein (LDL‐C) particles.^3^, ^4^ Following a diagnosis with CHD, secondary prevention with statins, antiplatelet and antihypertensive therapy is critical to reduce cardiovascular events and improve survival.^5^, ^6^ However, previous research documented gaps in hospital prescription of secondary prevention medications in Aboriginal and Torres Strait Islander people compared to non‐indigenous Australians.^7^ Following a diagnosis of CHD, regular and lifelong screening of CHD risk markers, namely diabetic and lipid markers, is required to guide up‐titration of medical therapy. While past research identified gaps, much of this work is more than a decade old and used hospital‐based rather than primary care records, where the majority of secondary prevention takes place.^8^, ^9^ We therefore aimed to determine differences in cardiovascular risk factors and comorbidities alongside differences in lipid and diabetes screening and secondary prevention medical therapy between Aboriginal and Torres Strait Islander people and non‐indigenous Australians with established CHD.

## Methods

This was an observational cohort study of the MedicineInsight^10^ database originally managed by the National Prescriber Service (NPS) MedicineWise Australia and more recently by the Australian Commission on Safety and Quality in Health Care. The MedicineInsight programme obtained electronic health records from all patient encounters at 662 general practices (primary care) with sampling from every Australian state and territory. Aboriginal community‐controlled health care organisations (ACCHCOs) were not included in the MedicineInsight database. MedicineInsight obtained data from all patients with at least one encounter from 1 January 2015 to 1 March 2021. In this cohort study, individuals were included if they were aged 18 years and over, had a recorded date of CHD diagnosis, received that diagnosis on or after their first clinical contact with the GP and were under active management by the GP (defined as three or more clinic visits in the 2 years prior).

This study was approved by the Research Integrity and Ethics Administration Human Research Ethics Committee (HREC) at the University of Sydney (number 2021/340). HREC seeks guidance on matters of research ethics involving Aboriginal and Torres Strait Islander peoples from Professor Jakelin Troy (on the manuscript) and other senior indigenous researchers at Sydney and from members of indigenous communities who sit on the committees. The study used de‐identified medical administrative data and was housed securely in the University of Sydney's research data store (RDS) to ensure safeguarding of the privacy and confidentiality of Aboriginal and Torres Strait Islander people's data. The study was conducted with engagement with Aboriginal and Torres Strait Islander people and researchers. The Aboriginal and Torres Strait Islander community was represented in our authors, and through them and their own community and indigenous research networks we benefitted from a deep practice‐based guidance on ethical community‐engaged research. Professor Sean Taylor is a Murray Island man and at the time was the Deputy Director of indigenous leadership and engagement and Executive Director of Aboriginal health and diversity at Northern Territory health. However, he is now the Director of Onemda: Aboriginal and Torres Strait Islander Health and WellBeing and Professor of Aboriginal and Torres Strait Islander Health at the University of Melbourne. Professor Jakelin Troy is Ngarigu of the Snowy Mountains in Southeastern Australia and is a professor of anthropology, director of indigenous research within the office of the deputy vice‐chancellor (research) and leader, indigenous research theme, the Charles Perkins Centre at the University of Sydney. Both have many decades of research with Aboriginal and Torres Strait Islander communities and guided the study in terms of direct value to community mindful of the CONSIDER framework^11^ and Sydney indigenous research strategy.^12^

The Bice–Boxerman index for continuity of care was used in the analysis as a covariate and is a measure of the extent to which a patient is loyal to one clinician within a clinic, which may change their health management.^13^ We determined whether a patient identified as an Aboriginal or Torres Strait Islander person by searching for the code ‘Aboriginal and/or Torres Strait Islander’, and for non‐indigenous Australians we searched ‘Neither Aboriginal and/or Torres Strait Islander’ within the patient's medical record. Individuals with no record of whether they were Aboriginal or Torres Strait Islander were removed from the analysis. CHD diagnosis was established based on coding with the term ‘coronary heart disease and atherosclerosis (CHD_ATH)’. This included limited coded non‐coronary types of atherosclerosis (e.g. AAA and occluded popliteal artery); however, the percentage of these was very small (<1%). Non‐coded documentation of CHD was also included, which comprised documentation of a diagnosis with ‘acute myocardial infarction’, ‘ST‐elevation myocardial infarction’, and ‘non‐ST‐elevation myocardial infarction’. Pathology records were used to ascertain screening for lipid and diabetes measurements. Lipid measurements included LDL‐C, HDL‐C, total cholesterol (TC) and triglycerides (TG), while diabetes mellitus was measured using a haemoglobin A1C (HbA1C) test. Systolic and diastolic blood pressure (BP) were taken from the medical records and, using the patients' most recent measurements, were categorised according to BP targets (<130/80 mmHg as per secondary prevention recommendations). Body mass index (BMI) was calculated from medical records of height and the median weight across an individual's follow‐up time. Smoking status was obtained from documentation in the medical records. Prescription data were used to identify cardiovascular medications with medications grouped into three categories: statins, angiotensin‐converting enzyme inhibitors (ACEI)/angiotensin II receptor antagonists (ARBs) and beta blockers. As aspirin is available over the counter, this secondary prevention medication could not be accurately identified, and therefore antiplatelet use was not analysed. Primary care was defined as health care management by a general practitioner.

The cohort was grouped into Aboriginal and Torres Strait Islander people and non‐indigenous Australians to compare the demographics, comorbidities and other medical information in a descriptive manner using t‐tests and chi‐squared tests. With such a large cohort, it was important to consider whether any statistically significant results were also clinically significant. Covariates with missing data were reported; however, in general all available medical records were used. The association of prescription of secondary prevention medications with whether they were Aboriginal and Torres Strait Islander people was assessed using logistic regression with a random effect for sites and adjusting for follow‐up time using the “glmer” function in R.^14^ The most parsimonious model was identified using least absolute shrinkage and selection operator (LASSO) method and then a binary variable of whether the patient was Aboriginal and Torres Strait Islander or non‐indigenous Australian was added to the model. A separate model was created for the outcomes of prescription of each cardiovascular medication class and for testing of lipids and HbA1c.

## Results

Our cohort included 50 088 patients with a diagnosis of CHD, treated in 406 GP clinics, with 1756 (3.5%) identifying as Aboriginal and Torres Strait Islander. Table 1 shows that Aboriginal and Torres Strait Islander people diagnosed with CHD were significantly younger, had higher female representation and were more likely to be current smokers and substance misusers, have higher BMI, have diabetes mellitus, chronic kidney disease and to live in outer regional, rural and remote areas of Australia. Aboriginal and Torres Strait Islander people were significantly more likely than non‐indigenous Australians to live in the most disadvantaged postcodes. There was no difference in the documentation of cardiovascular risk factors of diagnosed hypertension, dyslipidaemia or hypertriglyceridaemia between Aboriginal and Torres Strait Islander people and non‐indigenous Australians.

**Table 1:** Baseline characteristics of cohort with coronary heart disease: overall and by whether Aboriginal and Torres Strait Islander people

Characteristic	Total, *n* = 50 088	Aboriginal and Torres Strait Islander people, *n* = 1756	Non‐Indigenous, *n* = 48 332	*P*‐value
Demographic variables				
Female (missing *n* = 5) *n* (%)	19 516 (39.0%)	831 (47.3%)	18 685 (39.0%)	<0.001†
Age at diagnosis	65.9 (11.9)	57.1 (12.7)	66.2 (11.7)	<0.001‡
Remoteness (missing *n* = 182)				<0.001†
Major Cities, *n* (%)	28 422 (57.0%)	522 (30.0%)	27 900 (57.9%)	
Inner Regional, *n* (%)	13 393 (26.8%)	414 (23.8%)	12 979 (26.9%)	
Outer Regional, *n* (%)	7245 (14.5%)	719 (41.3%)	6526 (13.5%)	
Remote/Very remote, *n* (%)	846 (1.7%)	86 (4.9%)	760 (1.6%)	
IRSAD (2016) quintile (missing = 182)				<0.001†
Disadvantaged,1,2 *n* (%)	21 668 (43.4%)	984 (56.5%)	20 684 (42.9%)	
Middle, 3, *n* (%)	11 285 (22.6%)	455 (26.1%)	10 830 (22.5%)	
Advantaged, 4, 5, *n* (%)	16 953 (34.0%)	302 (17.3%)	16 651 (34.6%)	
Cardiac risk factors				
BMI^§^ (missing *n* = 6331)	28.1 (6.3)	29.3 (7.6)	28.1 (6.2)	<0.001‡
Smoking (missing *n* = 1478)				<0.001†
Current, *n* (%)	5254 (10.8%)	579 (33.9%)	4675 (10.0%)	
Past, *n* (%)	20 190 (41.5%)	652 (38.2%)	19 538 (41.7%)	
Never, *n* (%)	23 166 (47.7%)	478 (28.0%)	22 688 (48.4%)	
Any substance abuse, *n* (%)	2620 (5.2%)	258 (14.7%)	2362 (4.9%)	<0.001†
General practice carer interaction				
Years between diagnosis and last GP visit	5.9 (5.0)	5.6 (4.9)	5.9 (5.0)	0.004‡
Days between GP visits	39 (38)	39 (49)	39 (38)	>0.9‡
Continuity of care index	0.5 (0.2)	0.3 (0.2)	0.5 (0.2)	<0.001‡
Comorbidities				
Type 2 diabetes mellitus, *n* (%)	14 354 (28.7%)	849 (48.3%)	13 505 (27.9%)	<0.001†
Chronic kidney disease, *n* (%)	4016 (8.0%)	183 (10.4%)	3833 (7.9%)	<0.001†
Dyslipidaemia, *n *(%)	28 042 (56.0%)	993 (56.5%)	27 049 (56.0%)	0.6†
Hypercholesterolaemia, *n* (%)	16 750 (33.4%)	583 (33.2%)	16 167 (33.4%)	0.8†
Hyperlipidaemia, *n* (%)	9162 (18.3%)	317 (18.1%)	8845 (18.3%)	0.8†
Hypertriglyceridemia, *n *(%)	1293 (2.6%)	56 (3.2%)	1237 (2.6%)	0.10†
Hypertension, *n *(%)	33 783 (67.4%)	1160 (66.1%)	32 623 (67.5%)	0.2†
Peripheral vascular disease, *n *(%)	2542 (5.1%)	110 (6.3%)	2432 (5.0%)	0.021†
Rheumatic heart disease, *n *(%)	374 (0.7%)	40 (2.3%)	334 (0.7%)	<0.001†
Atrial fibrillation/flutter, *n* (%)	8626 (17.2%)	224 (12.8%)	8402 (17.4%)	<0.001†
Heart failure, *n* (%)	6547 (13.1%)	309 (17.6%)	6238 (12.9%)	<0.001†
Stroke or TIA, *n* (%)	5326 (10.6%)	185 (10.5%)	5141 (10.6%)	0.9†

† Pearson's chi‐squared test.

‡ Two sample t‐test.

§ Median across all follow‐up time.

BMI, body mass index; DBP, diastolic blood pressure; GP, general practice; HT, hypertension; IRSAD, index of relative socio‐economic advantage and disadvantage; SBP, systolic blood pressure.

**Figure 1:**
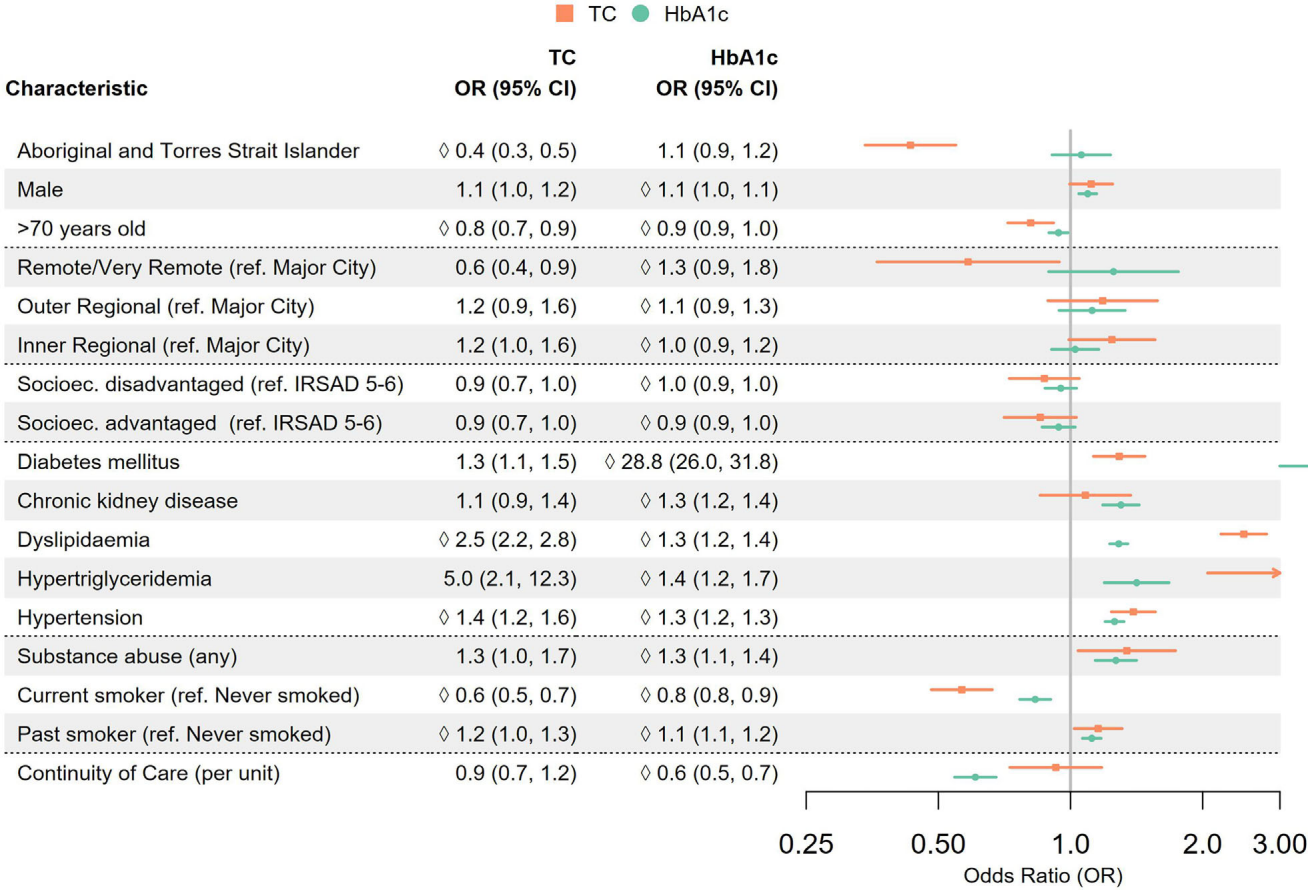
Forest plot of predictors of testing for total cholesterol (TC) and HbA1C in individuals with coronary heart disease.

The proportion of people in each group who underwent testing of lipid and diabetes markers is shown in Table 2. After a mean 5.9 years ±5.0 in primary care after their diagnosis with CHD, 7.5% of Aboriginal and Torres Strait Islander people and 3.5% of non‐indigenous Australians had no documentation of total cholesterol testing. From the parsimonious multivariable model (Fig. 1), Aboriginal and Torres Strait Islander people were significantly less likely to have lipids measured following a diagnosis with CHD (adjusted odds ratio (aOR) 0.4; 95% CI 0.3–0.5, *P* < 0.001) compared to non‐indigenous Australians. From patients who had pathology performed, LDL‐C targets of <1.8 were achieved in similar proportions of 35.2% versus 36.9% (*P* = 0.16 Table 2). Aboriginal and Torres Strait Islander people were less likely to meet HDL‐C or triglyceride targets, with HDL‐C >1.0 mmol/L (57.5% vs 73.7%, *P* < 0.001) and triglyceride <2 mmol/L (61.7% vs 76.0%, *P* < 0.001). Aboriginal and Torres Strait Islander people were more likely than non‐indigenous Australians to have their glucose measured (75.7% vs 66.6%, *P* < 0.001); however, a lower proportion had HbA1C ≤53 mmol/mol (≤7%), indicative of poorer glycaemic control (67.7% vs 82.1%, *P* < 0.001) (Table 2).

**Table 2:** Pathology tested, results and medication prescribed by whether Aboriginal and Torres Strait Islander people

Characteristic	Aboriginal and Torres Strait Islander people, *n* = 1756	Non‐Indigenous, *n* = 48 332	*P*‐value
Whether any tests documented			
BP not measured, *n* (%)	26 (1.5%)	546 (1.1%)	<0.001†
TC not measured, *n* (%)	132 (7.5%)	1669 (3.5%)	<0.001†
LDL‐C not measured, *n* (%)	241 (13.7%)	3064 (6.3%)	<0.001†
HDL‐C not measured, *n* (%)	221 (12.6%)	2762 (5.7%)	<0.001†
TRI not measured, *n* (%)	143 (8.1%)	1847 (3.8%)	<0.001†
HbA1c not measured, *n* (%)	426 (24.3%)	16 120 (33.4%)	<0.001†
BP measures			
SBPmm/Hg‡ (mean (SD))	130 (19)	133 (18)	<0.001§
DBPmm/Hg‡ (mean (SD))	77 (12)	75 (11)	<0.001§
Target BP¶, *n* (%)	641 (37.1%)	15 424 (32.3%)	<0.001†
HT category††, *n* (%)			
Normal	406 (23.5%)	8183 (17.1%)	<0.001†
Elevated	235 (13.6%)	7241 (15.2%)	
Hypertension Stage 1	738 (42.7%)	21 170 (44.3%)	
Hypertension Stage 2	351 (20.3%)	11 192 (23.4%)	
Lipid measures			
Total cholesterol (mean (SD))	4 31 (1.20)	4.24 (1.16)	0.02§
LDL‐C mmol/L			
Mean (SD)	2.28 (1.27)	2.22 (0.99)	0.20§
LDL‐C < 1.8 mmol/mol (7%), *n* (%)	533 (35.2%)	16 721 (36.9%)	0.16†
HDL‐C mmol/L			
Mean (SD)	1.15 (0.39)	1.30 (0.38)	<0.001§
HDL‐C >1 mmol/L, *n* (%)	883 (57.5%)	33 586 (73.7%)	<0.001†
TGs mmol/L			
Mean (SD)	2.10 (2.71)	1.61 (1.11)	<0.001§
TGs < 2 mmol/L, *n* (%)	883 (61.7%)	35 318 (76.0%)	<0.001†
HbA1c			
HbA1c ≤ 53 mmol/mol (7%), *n* (%)	900 (67.7%)	26 462 (82.1%)	<0.001§
Medications prescribed			
ACEI/ARBs	1146 (70.0%)	31 683 (69.0%)	0.4†
Beta blocker	551 (33.6%)	12 834 (27.9%)	<0.001†
Statins	1306 (79.7%)	37 312 (81.2%)	0.12†

† Pearson's chi‐squared test.

§ t‐test.

¶ BP Targets: BP < 130/80 mmHg.

†† Hypertension categories: Normal: SBP <120 AND DBP < 80, Elevated: SBP 120–129 AND DBP < 80, Hypertension Stage 1: SBP 130–139 OR DBP 80–8. Hypertension Stage 2: SBP≥140 OR DBP≥90.

ACEI, angiotensin‐converting enzyme inhibitors; ARB, angiotensin II receptor antagonists; BP, blood pressure; HbA1c, glycated haemoglobin; HDL‐C, high‐density lipoprotein‐cholesterol; LDL‐C, low‐density lipoprotein‐cholesterol; TC, total cholesterol; TGs, triglycerides.

The majority of patients had their BP measured, with 1.5% of Aboriginal and Torres Strait Islander people and 1.1% of non‐indigenous Australians having no record of BP being measured respectively. The recommended BP target (<130/80 mmHg) was achieved for 37.1% of Aboriginal and Torres Strait Islander people, significantly higher than the 32.3% observed in non‐indigenous Australians.

**Figure 2:**
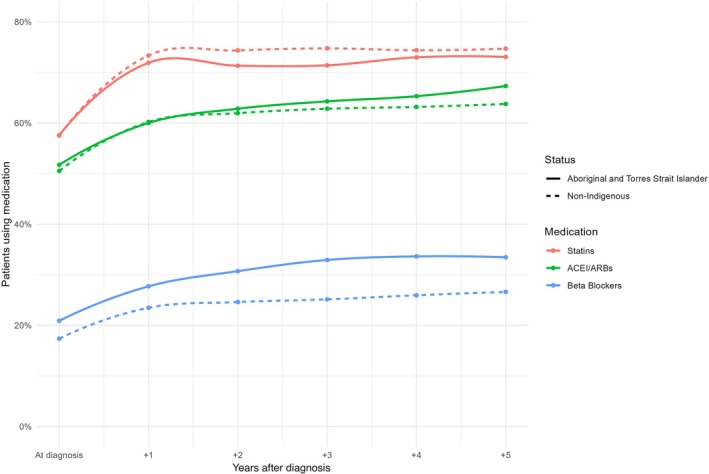
Proportion of people prescribed each cardiovascular medication class across time after coronary heart disease diagnosis.

**Figure 3:**
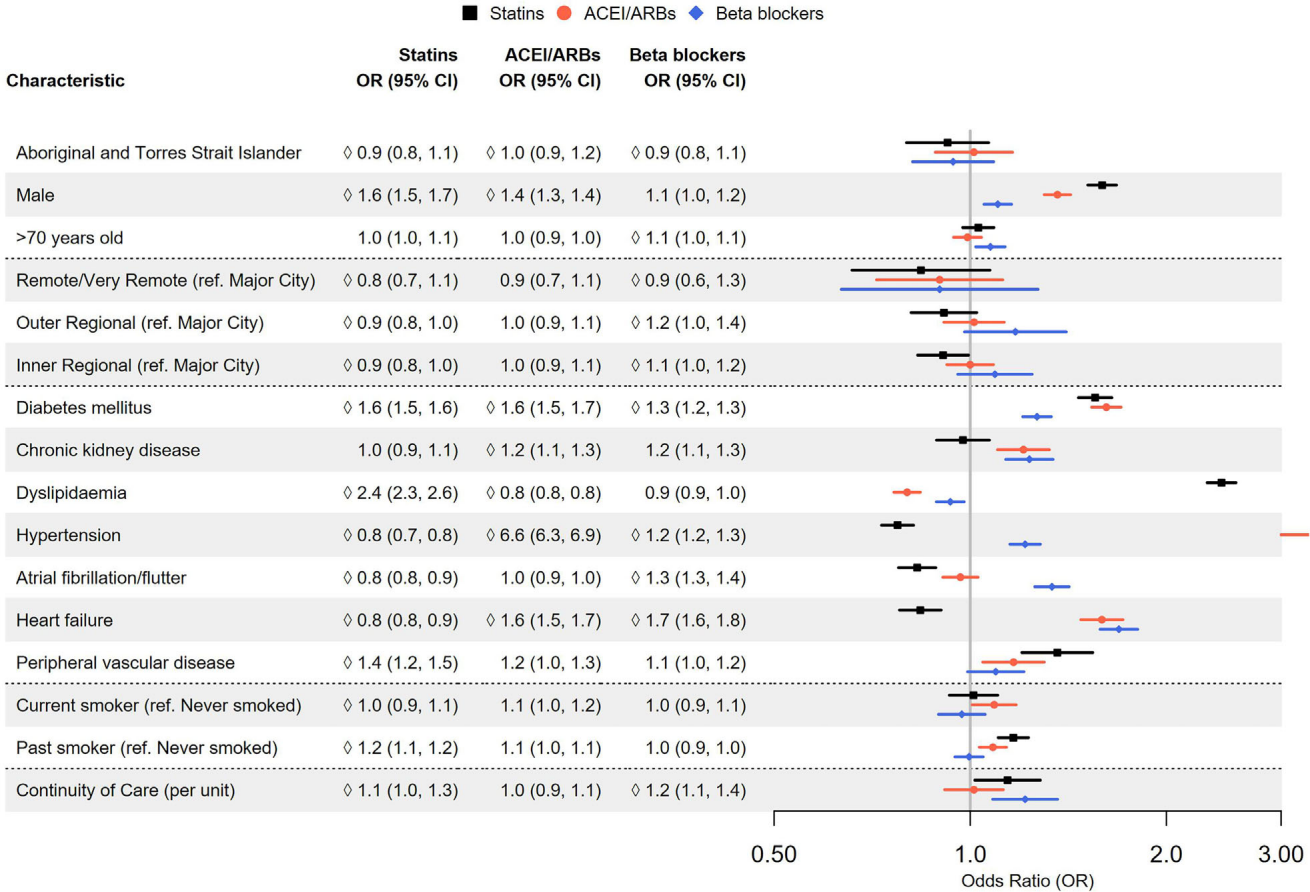
Forest plot of predictors of cardiovascular medication prescription in individuals with coronary heart disease.

Prescription of secondary prevention medications were similar for the two groups as shown in Table 2. The proportion prescribed secondary prevention medications remained at a similar level over time for Aboriginal and Torres Strait Islander people and non‐indigenous Australians, as shown in Figure 2. Beta blocker prescription was significantly higher in Aboriginal and Torres Strait Islander people than non‐indigenous Australians (33.6% vs 27.9%, respectively, *P* < 0.001). However, after adjustment in multivariate analysis, similar proportions of Aboriginal and Torres Strait Islander people, compared to non‐indigenous Australians with CHD were prescribed secondary prevention statins, ACE‐I/ARBs and beta blockers (Fig. 3). All the variables in Figure 3 were significant in the parsimonious multivariable model, namely gender, age, remoteness, socioeconomic status, comorbidities, smoking, substance misuse and continuity of care.

## Discussion

This nation‐wide study of Australian people with CHD treated in primary care demonstrates a marked difference in cardiovascular risk factor profile such as BMI, smoking and comorbidity burden among Aboriginal and Torres Strait Islander people compared to non‐indigenous Australians. Most striking was the 9.1‐year age gap at the time of CHD diagnosis, but also notable is the higher female representation, higher proportion with type 2 diabetes mellitus diagnosis and renal failure, alongside sociodemographic differences including a higher proportion currently smoking, more living in rural/remote and more from lower socioeconomic areas. Importantly, no gap was found in the prescription of secondary prevention medications such as statins and antihypertensives, and Aboriginal and Torres Strait Islander people were just as likely to meet BP and LDL‐C targets and more likely to have had a HbA1C test. However, metabolic syndrome markers, characterised by low HDL‐C, high triglycerides and diabetes mellitus, as well as active smoking, were prominent in Aboriginal and Torres Strait Islander people with CHD and potential targets to reduce residual cardiovascular risk.

Consistent with previous research, including among the Māori, Pacific and Indian people of New Zealand,^15^ we found that CHD occurred almost a decade earlier in Aboriginal and Torres Strait Islander people, with a higher burden of cardiovascular risk factors,^16^, ^17^, ^18^, ^19^ including higher BMI, current smoking, diabetes mellitus, chronic renal failure and substance misuse. This suggests that prematurity of CHD in Aboriginal and Torres Strait Islander people may reflect a higher burden of certain cardiovascular risk factors, coupled with differences in social determinants of health.^20^ In order to equitably treat CHD in Australia, more needs to be done to ensure care meets the needs of Aboriginal and Torres Strait Islander people^21^, ^22^, ^23^ and that complies with the philosophy of the Aboriginal Community Controlled Health Organisations stated principle that “*Aboriginal Health means not just the physical well‐being of an individual but refers to the social, emotional and cultural well‐being of the whole Community in which each individual is able to achieve their full potential as a human being*.”

This study did not find a difference in the proportion of Aboriginal and Torres Strait Islander people compared to non‐indigenous Australians who met secondary prevention targets for key measures of LDL cholesterol, BP and prescription of secondary prevention medications. It suggests that Aboriginal and Torres Strait Islander people with CHD who regularly attend primary care appear to be achieving CVD risk factor targets. This is in conflict with other research that found Aboriginal and Torres Strait Islander people were less likely to have CVD risk screening^2^ and less likely to be on preventive medications after being discharged from hospital after angiography^24^ or to receive appropriate discharge medications.^25^ It is possible that our study represents a population more likely to receive appropriate secondary prevention care, as inclusion criteria included only patients with a known diagnosis of CHD who regularly attended primary care.

In people with CHD, regular screening of lipids and HbA1C is crucial to determine if secondary prevention targets are being met, and to guide up‐titration of medical therapy and lifestyle changes. We found that Aboriginal and Torres Strait Islander people had a higher proportion of testing of HbA1c, which may reflect the higher burden of diabetes mellitus and/or be related to HbA1C point of care testing in rural communities. This study found that Aboriginal and Torres Strait Islander people with CHD were significantly less likely to have their lipids measured, even after adjustment for confounders, compared with non‐indigenous Australians. This was after extended follow‐up of 6 years on average and in people who by definition were under regular care by their general practitioner. While the proportion of people who met secondary prevention targets for BP and LDL‐C were similar between Aboriginal and Torres Strait Islander people and non‐indigenous Australians, there were marked differences in metabolic syndrome markers with elevations in triglycerides and HbA1C, alongside low HDL‐C, consistent with previous research.^3^, ^4^, ^26^ This is important, as this ‘residual’ cardiovascular risk is likely to contribute to the higher CHD‐related deaths seen in Aboriginal and Torres Strait Islander people and provide targets for future interventions. Such interventions would need to consider social determinants of health, for which we also found significant differences with Aboriginal and Torres Strait Islander people more likely to be living in rural/remote areas and in areas of lower socioeconomic status compared to non‐indigenous Australians. These may impact on accessibility to health care as well as health literacy, all important factors in the design of interventions.

This study was limited by the following: Only patients that regularly attended their GP were included, which may have created a selection bias, with the potential for larger gaps in care seen in patients who do not regularly attend a primary care practice. Medical record data were used, and due to the large sample size, statistical significance must be interpreted carefully in the context of clinically meaningful differences. Our study reported on prescription of medications and cannot inform on individual adherence/compliance with this prescription. Further, continuity of care, namely visiting the same general practitioner, was significantly higher in non‐indigenous Australians, although the time gap between general practitioner visits was similar. A total of 3.5% of our cohort of patients with CHD were Aboriginal and Torres Strait Islander people, which is marginally lower than the national average of 3.8%.^27^ This may reflect the selection bias of only including people who regularly attended their GP and/or reflect the fact that Aboriginal and Torres Strait Islander people with CHD may have been more often managed within ACCHCOs, which were not included in our dataset.

## Conclusions

This large‐scale analysis of primary care data of people with CHD from all Australian states and territories offers important insights into Aboriginal and Torres Strait Islander people's health. We found differences in underlying cardiovascular risk factors and comorbidities including in socioeconomic determinants of health, alongside differences in lipid profiles, glucose levels and smoking patterns. Our findings suggest that when Aboriginal and Torres Strait Islander people are engaged regularly in primary care, some significant goals of CHD secondary prevention are achieved. Further insight and solutions enabling regular access to primary care may be facilitated through utilising bidirectional learning between the current, predominantly a non‐indigenous primary care paradigm and ACCHCOs.
